# Tetraphenylethene-Embedded Pillar[5]arene and [15]Paracyclophane: Distorted Cavities and Host–Guest Binding Properties

**DOI:** 10.3390/molecules26195915

**Published:** 2021-09-29

**Authors:** Yingtao Fan, Kaitai Hu, Junyi Nan, Yingzhong Shen

**Affiliations:** Applied Chemistry Department, College of Material Science & Engineering, Nanjing University of Aeronautics & Astronautics, Nanjing 210016, China; fanyt@nuaa.edu.cn (Y.F.); hkt123666@163.com (K.H.); junyi0519@outlook.com (J.N.)

**Keywords:** pillararene, [15]Paracyclophane, aggregation-induced emission, meso functionalization, host–guest

## Abstract

Two aggregation-induced emission (AIE) macrocycles (**DMP[5]-TPE** and **PCP[5]-TPE**) were prepared by embedding Tetraphenylethene (TPE) unit into the skeletons of Dimethoxypillar[5]arene (DMP[5]) and [15]Paracyclophane ([15]PCP) at meso position, respectively. In crystal, the **PCP[5]-TPE** showed a distorted cavity, and the incubation of hexane inside the **DMP[5]-TPE** cavity caused a distinct change in the molecular conformation compared to **PCP[5]-TPE**. There was no complexation between **PCP[5]-TPE** and 1,4-dicyanobutane (DCB). UV absorption experiments showed the distorted cavity of **DMP[5]-TPE** hindered association with DCB.

## 1. Introduction

Macrocyclic molecules are the most important supramolecular hosts, with multivalent binding sites and ring-like structures [1]. Pillararenes are new generation macrocyclic molecules, made up of hydroquinone units linked by methylene bridges [2,3,4,5]. The structures of pillararenes are similar to that of calixarenes, but are much more symmetrical [1]. Pillararenes show excellent host–guest binding properties, which can not only recognize ionic guests [6,7], but also interact strongly with selective neutral objects [8,9]. [15]Paracyclophane ([15]PCP) received a great deal of attention since the first report by Huang in 2018 [10]. [15]PCP is a new carbon-bridged macrocycle, and its structure is similar to that of pillar[5]arene. [15]PCP can be synthesized by the hydrodeoxygenation of pillar[5]arene via aryl fluorosulfonate intermediates with a high yield.

There are several potential modification positions in pillararene, such as rims [4,11,12,13,14], meta, and bridging meso positions [15]. Current explorations of function pillararene still focus on the meta or rim positions. However, the functionalization of pillararene at the meso position has not been as extensively explored as that of their meta or rim positions. Meanwhile, due to the absence of the alkoxy groups, there are fewer modification sites than pillararenes, so the modification of the meso position of [15]PCP is particularly important [16]. Unlike the rim positions, the modifications at the meso positions may distort the cavity of the pillararene, which depends on the structure of the introduced substituents [15,17]. Meanwhile, a distorted cavity may exhibit different host–guest binding properties. Therefore, it is important to explore the host–guest binding properties of the meso position of pillararene.

Tetraphenylethylene (TPE) is an aggregation-induced emission (AIE) molecule that has been extensively studied. The TPE skeleton owns propeller-like conformation with one double-bond stator and four phenyl rotors [18,19,20,21,22]. The TPE unit can be embedded into the skeletons of pillararenes by a few reactions. To maintain the lower-energy state of the TPE unit, the skeletons of pillararene are deformed to keep their propeller-like conformation [15].

## 2. Results and Discussion

In this research, we report two macrocycles (**DMP[5]-TPE** and **PCP[5]-TPE**) prepared by embedding the TPE unit into the meso position of the skeletons of Dimethoxypillar[5]arene (DMP[5]) and [15]PCP, respectively.

**Scheme 1:**
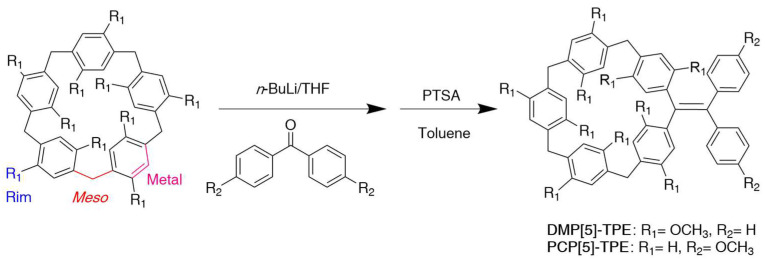
Synthesis of **DMP[5]-TPE** and **PCP[5]-TPE**. PTSA = *p*-toluenesulphonic.

As shown in Scheme 1, DMP[5] the meso position anion was generated by deprotonation of DMP[5] with 5.0 eqiv. of *n*-butyllithium. Then, the DMP[5] anion proceeded to the nucleophilic addition reaction with 6.0 equiv. of benzophenone, which was followed by dehydration using *p*-toluenesulphonic acid as a catalyst in the refluxing toluene, resulting in **DMP[5]-TPE** with an overall yield of 6.7% (Figures S1–S4). The synthesis of **PCP[5]-TPE** was carried out in the same way as for the synthesis of **DMP[5]-TPE**, but [15]PCP was used instead of DMP[5], and 4,4’-dimethoxybenzophenone instead of benzophenone, resulting in **PCP[5]-TPE** with an overall yield of 95% (Figures S5–S8). Unexpectedly, under the same experimental procedures, the yield of **DMP[5]-TPE** was much lower than **PCP[5]-TPE**. Two main reasons may account for this significant difference: (1) In common solvents for lithiation reactions, DMP[5] is only slightly more soluble in THF, and so a large amount of undissolved DMP[5] could not participate efficiently in the lithiation reaction. Contrarily, [15]PCP has good solubility in THF. (2) The structure of DMP[5] contains ten methoxy groups, and these electron-donating groups could increase the electron density of the meso position and decrease the lithiation reaction yield. The identities of the above-mentioned products were all unequivocally confirmed by NMR spectroscopy and high-resolution mass spectroscopy.

Take the ^1^H NMR of **DMP[5]-TPE** as an example (Figure S1). The resonance at δ = 7.02 ppm as a single peak was assigned to the phenyl protons of the TPE unit outside the skeleton of pillar[5]arene. The resonance peaks of Δ = 6.72, 6.66, 6.65, 6.36 and 6.18 ppm were assigned to the phenyl protons of the pillar[5]arene, the δ = 3.81 and 3.72 resonance peaks were assigned to the remaining meso protons, and the δ = 3.66, 3.61, 3.54, 3.38, and 2.65 ppm resonance peaks were assigned to the methoxy groups protons. Remarkably, the resonance of the six protons of the methoxy groups at δ = 2.65 ppm as a single peak showed a high-field shift, which indicated that the two methoxy groups rotated in the cavity of the pillar[5]arene due to the propeller-like conformation of the TPE unit.

**Figure 1:**
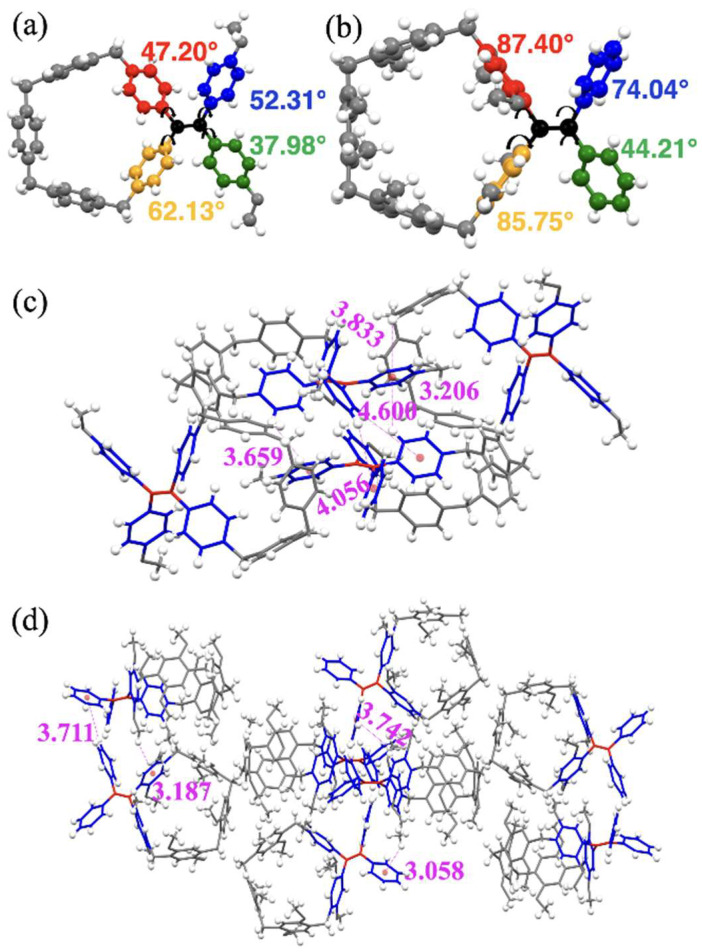
Top view of crystal structures **PCP[5]-TPE** and **DMP[5]-TPE** (**a**,**b**) and molecular packing of **PCP[5]-TPE** and **DMP[5]-TPE** (**c**,**d**), Hexane solvent molecules are omitted for clarity.

Fortunately, the crystal of **DMP[5]-TPE** and **PCP[5]-TPE** was obtained by slowly evaporating the mixed solutions of dichloromethane and petroleumether. As shown in Figure 1, the TPE unit was embedded in the skeletons of the pillararenes successfully. With no complexation with the guest, the pillar scaffold of **PCP[5]-TPE** adopts an exceedingly distorted conformation (Figure 1a and Figure S9). The dihedral angles between the phenyl moieties of the TPE unit and the ethylene plane are 37.98°, 47.2°, 52.31°, and 62.13°, respectively. In contrast, due to the complexation with hexane, **DMP[5]-TPE** shows an almost symmetrical cavity identical to that of DMP[5] (Figure 1b and Figure S11). The dihedral angles between the phenyl groups and the planes constructed by the five-pillar meso carbons are 44.21°, 74.04°, 87.40°, and 87.75°, respectively. These results indicate that the combination of host–guest will induce a significant change in the molecular conformation.

**PCP[5]-TPE** crystalizes in a monoclinic *P*2_1_/*n* space group. Short ArH- contacts exist between the adjacent TPE units (Figure 1c and Figure S10). These intermolecular contacts make the molecules more tightly connected and further restrict the intramolecular movement of the TPE units, leading to the intense fluorescence in the polymerized state. Further measurements of the molecular arrangements revealed the intermolecular ArH- interaction with a minimum contact distance of 3.206 Å. The same study for **DMP[5]-TPE** showed that **DMP[5]-TPE** crystalized in an orthorhombic *Pbca* space group, and that there was an intermolecular ArH- interaction with a minimum contact distance of 3.058 Å (Figure 1d and Figure S12). Therefore, **DMP[5]-TPE** is packed more tightly than **PCP[5]-TPE**.

**Figure 2:**
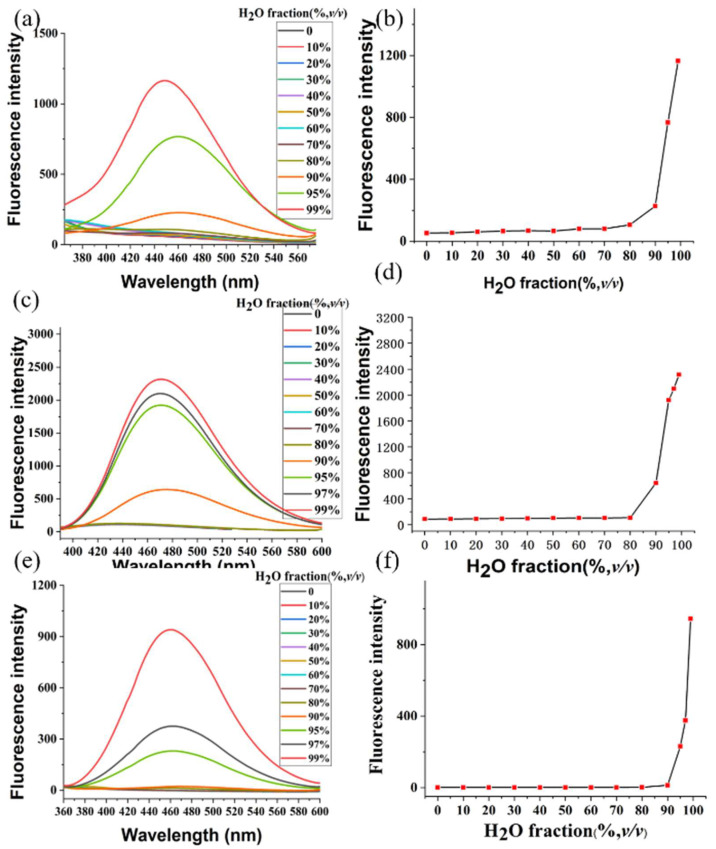
Fluorescence spectra of **DMP[5]-TPE** (**a**,**b**), **PCP[5]-TPE** (**c**,**d**) and **TPE** (**e**,**f**) in different THF/H_2_O mixed solutions. (Dilute solutions of 1 × 10^−5^ mol/L **DMP[5]-TPE**, **PCP[5]-TPE** and TPE in THF excitation at 300 nm).

The fluorescent emission properties of **DMP[5]-TPE** and **PCP[5]-TPE** were investigated in the THF–H_2_O mixture at excitation of 300 nm. As shown in Figure 2, dilute solution of 1 × 10^−5^ mol/L **DMP[5]-TPE** in THF still presented a weak fluorescence emission, and the dilute solution of **PCP[5]-TPE** in THF emitted no light. This was most likely due to the steric hindrance exerted by the methoxy groups in the TPE unit embedded in the skeleton of **DMP[5]-TPE**, which leads to a greater restriction on the TPE phenyl groups’ rotational freedom. With the gradual addition of water into the dilute solution of **DMP[5]-TPE** in THF, when the *f*_w_ (the volume percentage of H_2_O in the THF–H_2_O mixture) was below 70%, the fluorescence emission remained almost unchanged in profile. However, when *f*_w_ increased to over 80%, the fluorescence intensity significantly increased (Figure 2a,b, Figures S13 and S14). Meanwhile, the **PCP[5]-TPE** solution in the THF–H_2_O mixture displayed a significant enhancement in fluorescence when *f*_w_ exceeded 90% (Figure 2c,d, Figures S15 and S16). In addition, in terms of the fluorescence wavelength, with the *f*_w_ increasing from 90% to 99%, the aggregation-induced fluorescence of **DMP[5]-TPE** showed a 12 nm blue shift from 460.2 nm to 448.2 nm. At the same time, **PCP[5]-TPE** showed a 3.4 nm blue shift from 475 nm to 471.6 nm. In the same experiments for free TPE, a remarkable fluorescence enhancement was observed when *f*_w_ increased to 95% (Figure 2e,f, Figures S17 and S18). The *f*_w_ increase from 95% to 99% was accompanied by a blue shift in the emission wavelength of 1.1 nm from 462.3 nm to 460.2 nm. The earlier fluorescence emission enhancement of **DMP[5]-TPE** and **PCP[5]-TPE** compared to free TPE suggested that the pillararene structure strongly restricted the rotational freedom of the TPE phenyl groups.

**Figure 3:**
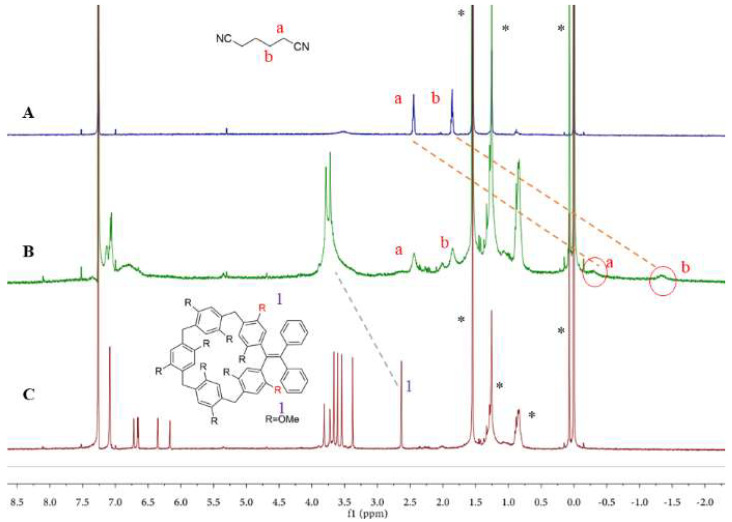
(**A**) ^1^H NMR spectra of DCB (1 mM), (**B**) Partial ^1^H NMR spectra of the mixed solution of **DMP[5]-TPE** (1 mM) and DCB (1 mM), (**C**) ^1^H NMR spectra of **DMP[5]-TPE** (1 mM). * = solvent.

**Figure 4:**
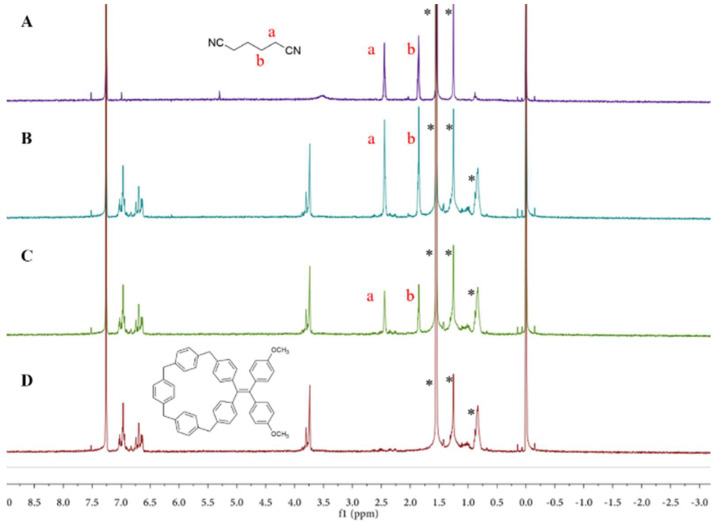
(**A**) ^1^H NMR spectra of DCB (1 mM), (**B**) Partial ^1^H NMR spectra of the mixed solution of **PCP[5]-TPE** (1 mM) and DCB (1 mM), (**C**) Partial ^1^H NMR spectra of the mixed solution of **PCP[5]-TPE** (1 mM) and DCB (2 mM), (**D**) ^1^H NMR spectra of **PCP[5]-TPE** (1 mM). * = solvent.

Subsequently, the binding properties of **DMP[5]-TPE** and **PCP[5]-TPE** were first performed by ^1^H NMR (Figure 3, Figure 4, Figure S19 and S20). As the guest for the host–guest interaction research, 1,4-dicyanobutane (DCB) was selected. As shown in Figure 3, The ^1^H NMR spectra of the solution of **DMP[5]-TPE** in CDCl_3_ in the absence and presence of the DCB guest were recorded. For the complex, on the NMR timescale, a slow exchange was observed. The resonances of the new species are consistent with the formation of an interpenetrated complex. In the presence of 1.0 equivalent DCB, the five resonance peaks of the phenyl protons of the pillar[5]arene skeleton were merged into a slope-shaped resonance peak accompanied by a minor low-field shift. At the same time, the resonance assigned to phenyl protons of the TPE unit outside the skeleton of pillar[5]arene were shown as multiplet peaks. The remaining meso protons and methoxy groups protons of the pillararene showed a double peak signal. Especially, the disappearance of the signal of the methoxy groups at δ = 2.65 ppm suggested that the distortion of the cavity of **DMP[5]-TPE** decreased and the cavity became slightly symmetric. Due to the inclusion-induced shielding effects with the presence of **DMP[5]-TPE**, the peaks of the methylene protons of the DCB showed a high-field shift and broadening effects (Δδ = −2.75 and −3.2 ppm for H_a_ and H_b_). The broadening and field shift of the resonance peaks of **DMP[5]-TPE** and the DCB mixtures indicated that the host, **DMP[5]-TPE** (wheel), was threaded by guest G (axle). **DMP[5]-TPE** and DCB could form an inclusion host–guest complex.

Unexpectedly, for the **PCP[5]-TPE**, as shown in Figure 4, even with the addition of two equivalents of DCB, the peaks of **PCP[5]-TPE** and DCB showed no observable shifts or broadening. This phenomenon indicated that no complexation, or weak complexation between **PCP[5]-TPE** and DCB, could be attributed to the absence of the methoxy groups in the [15]PCP structure because the dipole–dipole interaction between the methoxy groups and DCB played a leading role in the host–guest binding [1,23].

The stoichiometry of complexation between **DMP[5]-TPE** and DCB was further investigated by Job’s plot method based on the UV-vis absorption experiments (Figure S21). Which indicated the 1:1 binding stoichiometry between **DMP[5]-TPE** and DCB. Furthermore, the ESI–MS spectrum of **DMP[5]-TPE** and DCB equimolar mixture showed one intense peak at *m/z* 1022.46 for the 1:1 host–guest complex ([**DMP[5]-TPE** + DCB]^+^), which also indicated the formation of 1:1 host–guest complex between **DMP[5]-TPE** and DCB (Figure S22). In order to determine the association constant for DCB ⊂ **DMP[5]-TPE** in CHCl_3_, UV-Vis titration experiments with a constant concentration of **DMP[5]-TPE** and varying concentrations of DCB were carried in CHCl_3_. The association constant (*K*_a_) of DCB ⊂ **DMP[5]-TPE** was determined to be (1.33 ± 0.05) × 10^4^ M^−1^ (Figure S23). In order to investigate the changes of the host–guest binding properties of the meso position of pillararene, DMP[5] was also tested in the same manner. The association constant (*K*_a_) of DCB ⊂ DMP[5] was determined to be (2.14 ± 0.08) × 10^4^ M^−1^ (Figure S24). The association constants for DCB ⊂ **DMP[5]-TPE** and DCB ⊂ DMP[5] were both in the vicinity of 10^4^ M^−1^ in CHCl_3_, and DCB ⊂ **DMP[5]-TPE** was slightly lower. The ^1^H NMR spectra of the solution of **DMP[5]-TPE** in CDCl_3_ in the presence of the DCB guest showed the re-symmetry of the cavity of **DMP[5]-TPE**. Thus, DCB was required to overcome the hindrances of the distorted cavity during the host–guest binding process. This could possibly be the reason for the decreased association constant for **DMP[5]-TPE** compared to the DMP[5] (Table S1).

## 3. Conclusions

In conclusion, we synthesized two AIE macrocycles (**DMP[5]-TPE** and **PCP[5]-TPE**) by embedding the TPE unit into the skeletons of DMP[5] and [15]PCP at meso position, respectively. **DMP[5]-TPE** and **PCP[5]-TPE** both showed a typical AIE nature. A crystal structure analysis revealed the distortion of the **PCP[5]-TPE** cavity, which maintained the lower-energy state of TPE unit, while the **DMP[5]-TPE** cavity was nearly symmetric due to the complexation with the guest. The ^1^H NMR spectra of the solution of **DMP[5]-TPE** CDCl_3_ in the presence of the DCB guest indicated the host–guest binding between **DMP[5]-TPE**. Conversely, **PCP[5]-TPE** could not form the host–guest complex with DCB, which was attributed to the absence of alkoxy groups in [15]PCP structure. The association constant for DCB ⊂ **DMP[5]-TPE** in CHCl_3_ was lower than DCB ⊂ DMP[5], due to the hindrances of the distorted cavity.

## Data Availability

Not applicable.

## Supplementary Materials

The following are available online. Figure S1: ^1^H NMR spectrum of **DMP****[5]-TPE** in CDCl_3_, Figure S2: ^13^C NMR spectrum of **DMP****[5]-TPE** in CDCl_3_, Figure S3: HRMS data for **DMP[5]-TPE** (Calculated [M + H]^+^: C_58_H_59_O_10_^+^ = 915.4103, Found: 915.3973), Figure S4: HRMS data for **DMP[5]-TPE** (Calculated [M + Na]^+^: C_58_H_58_NaO_10_^+^ = 937.3922, Found: 937.3965), Figure S5: ^1^H NMR spectrum of **PCP****[5]-TPE** in CDCl_3_, Figure S6: ^13^C NMR spectrum of **PCP****[5]-TPE** in CDCl_3_, Figure S7: HRMS data for **PCP[5]-TPE** (Calculated [M + Na]^+^: C_50_H_42_NaO_2_^+^ = 697.3077, Found: 697.3122), Figure S8: HRMS data for **PCP[5]-TPE** (Calculated [M]^+^: C_50_H_42_O_2_^+^ = 674.3179, Found: 674.3195), Figure S9: Top (a) and side view (b) of crystal structure **PCP****[5]-TPE**, Figure S10: Molecular packing of **PCP****[5]-TPE**, Figure S11: Top (a) and side view (b) of crystal structure **DMP[5]-TPE**. Hexane solvent molecules are omitted for clarity, Figure S12: Molecular packing of **DMP[5]-TPE**. Hexane solvent molecules are omitted for clarity, Figure S13: Fluorescence spectra of **DMP****[5]-TPE** (1 × 10^−5^ M), Figure S14: Plot of the highest fluorescence intensity in different THF/H_2_O mixed solution of **DMP****[5]-TPE**, Figure S15: Fluorescence spectra of **PCP****[5]-TPE** (1 × 10^−5^ M), Figure S16: Plot of the highest fluorescence intensity in different THF/H_2_O mixed solution of **PCP****[5]-TPE**, Figure S17: Fluorescence spectra of **PC****[5]-TPE** (1 × 10^−5^ M), Figure S18: Plot of the highest fluorescence intensity in different THF/H_2_O mixed solution of **PCP****[5]-TPE**, Figure S19: (A) ^1^H NMR spectra of DCB (1 mM), (B) Partial ^1^H NMR spectra of mixed solution of **DMP****[5]-TPE** (1 mM) and DCB (1 mM), (C) ^1^H NMR spectra of **DMP****[5]-TPE** (1 mM). * = solvent, Figure S20: (A) ^1^H NMR spectra of DCB (1 mM), (B) Partial ^1^H NMR spectra of mixed solution of **PCP****[5]-TPE** (1 mM) and DCB (1 mM), (C) Partial ^1^H NMR spectra of mixed solution of **PCP****[5]-TPE** (1 mM) and DCB (2 mM), (D) ^1^H NMR spectra of **PCP****[5]-TPE** (1 mM). * = solvent, Figure S21: Job plot showing the 1: 1 stoichiometry of the complex between **DMP****[5]-TPE** and DCB in CHCl_3_ by plotting the Δδ in absorbance of the 280nm observed by uv-vis against the mole fraction of guest (X_guest_). ([host] + [guest] = 2 × 10^−5^ M, Figure S22: ESI-MS spectrum of the host−guest complex formed between **DMP****[5]-TPE** and DCB. (Calculated [**DMP****[5]-TPE** + DCB]^+^: C_64_H_66_N_2_O_10_^+^ = 1022.4712, Found: 1022.4657), Figure S23: (a) The absorbance of **DMP****[5]-TPE** and DCB mixture. (b) The Red line was obtained from the nonlinear curve-fitting (*K*_a_ = ((1.33 ± 0.05) × 10^4^ M^−1^, R^2^ = 0.9923), Figure S24: (a) The absorbance of DMP[5] and DCB mixture. (b) The Red line was obtained from the nonlinear curve-fitting (*K*_a_ = ((2.14 ± 0.08) × 10^4^ M^−1^, R^2^ = 0.9928), Table S1: Association constant for DMP[5], **DMP[5]-TPE** and **PCP[5]-TPE**.

## Sample Availability

Not available.
