# Supplementary material for: Tetraphenylethene-Embedded Pillar[5]arene and [15]Paracyclophane: Distorted Cavities and Host–Guest Binding Properties
Source: Molecules. 2021 Sep 29;26(19):5915. doi: 10.3390/molecules26195915 (PMC8512412; doi:10.3390/molecules26195915)
Supplement: Supplementary file 1 [file molecules-26-05915-s001.zip › molecules-1381000-supplementary.pdf]

Electronic Supplementary Information

Tetraphenylethene embedded pillar[5]arene and [1_5]Paracyclophane: distorted cavities and host-guest binding properties

Yingtao Fan, Kaitai Hu, Junyi Nan, Yingzhong Shen*

Applied Chemistry Department, College of Material Science & Engineering, Nanjing University of Aeronautics & Astronautics, Nanjing 210016, P. R. China

Table of Contents

1. **General information**
2. **Synthesis and characterization of DMP[5]-TPE**
3. **Synthesis and characterization of PCP[5]-TPE**
4. **X-ray crystallography data of PCP[5]-TPE**
5. **X-ray crystallography data of DMP[5]-TPE**
6. **Fluorescence spectra of DMP[5]-TPE and PCP[5]-TPE**
7. **Binding experiment with 1,4-dicyanobutane of DMP[5]-TPE and DMP[5]**

1. General information

[1_5]PCP[1] was synthesized according to previously reported method. All reactions were performed under nitrogen atmosphere unless otherwise stated. THF was distilled from Na/benzophenone ketyl prior to use, and other reagents were commercially available and used as received. NMR spectra were recorded with a Bruker AVANCE 400 spectrometer at resonant frequencies of 400 MHz for ^{1}H and 101 MHz for ^{13}C nuclei using CDCl3 as deuterated solvent. Fluorescence spectra were recorded on a Gangdong SCI F-380 Fluorescence spectrophotometer excitation at 300 nm. UV-visible spectra were recorded on Shimadzu UV 1780 UV-Vis Spectrophotometer. High-resolution electrospray ionization mass spectra (HR-ESI-MS) were recorded on an Agilent 6540Q-TOF LCMS equipped with an electrospray ionization (ESI) probe operating in positive-ion mode with direct infusion. The analyses of hydrogen and carbon were carried out by direct combustion on a Perkin-Elmer 240C element analyzer.

2. Synthesis and characterization of **DMP[5]-TPE**

The synthesis of **DMP[5]-TPE** was adapted from a previously reported method. To a suspension of DMP[5] (488 mg, 0.65 mmol) in anhydrous THF (47.5 mL), 2.4 M solution of *n*-butyllithium in hexane (1.35 mL, 3.24 mmol) was added at 0˚C under nitrogen. Afterwards, the reaction mixture was gradually brought back to room temperature (25˚C) and stirred 1 h. Benzophenone (710 mg, 3.9 mmol) was degassed and added to the reaction solution under nitrogen. The reaction temperature was warmed to 70˚C and strirred overnight. The recation quenched by adding H_2O (1 mL). Subsequently, the solvent was evaporated under vacuum, offering red-orange oily liquid. The obtained oily liquid was dissolved in CH_2Cl_2 (50 mL), and washed with 1M HCl (10 mL) and H_2O (2 × 30 mL), dried over anhydrous Na_2SO_4 and concentrated. The concentrate was diluted with CH_2Cl_2 (1 mL) and slowly added dropwise to the vigorously stirred CH_3OH (50 mL). The solid immediately precipitated, suction filtrated, and dried to afford pale-yellow solid.

The resulting solid was dissolved in toluene (60 mL), *p*-toluenesulfonic acid (190 mg) was added and the reaction mixture was refluxed for 6 h using a Dean-Stark apparatus. The toluene was evaporated under vacuum, offering oily liquid. The obtained oily liquid was dissolved in CH_2Cl_2 (50 mL), washed with saturated Na_2CO_3 solution (20 mL) and H_2O (30 mL), dried over anhydrous Na_2SO_4 and concentrated. Pure **DMP[5]-TPE** (40 mg, 6.7%) was obtained by column chromatography (PE/dichloromethane = 4/1, *v*/*v*). ^{1}H NMR (400 MHz, $CDCl_3$, 298 K)) δ 7.08 (s, 10H), 6.72 (s, 2H), 6.66 (d, J = 5.8 Hz, 4H), 6.36 (s, 2H), 6.18 (s, 2H), 3.81 (s, 4H), 3.72 (s, 4H), 3.66 (s, 6H), 3.54 (s, 6H), 3.38 (s, 6H), 2.65 (s, 6H). ^{13}C NMR (101 MHz, $CDCl_3$, 298 K) δ 151.81, 151.38, 151.27, 151.24, 151.06, 143.78, 141.72, 131.82, 131.07, 129.94, 129.35, 129.05, 128.23, 127.67, 126.52, 115.74, 115.52, 114.85, 114.34, 114.15, 56.55, 56.32, 56.26, 56.16, 56.09, 53.71, 30.03, 30.01, 29.78, 29.67. Anal. Calcd. for $C_{58}H_{58}O_{10}$: H, 6.98; C, 76.13, Found: H, 6.76; C, 75.84.

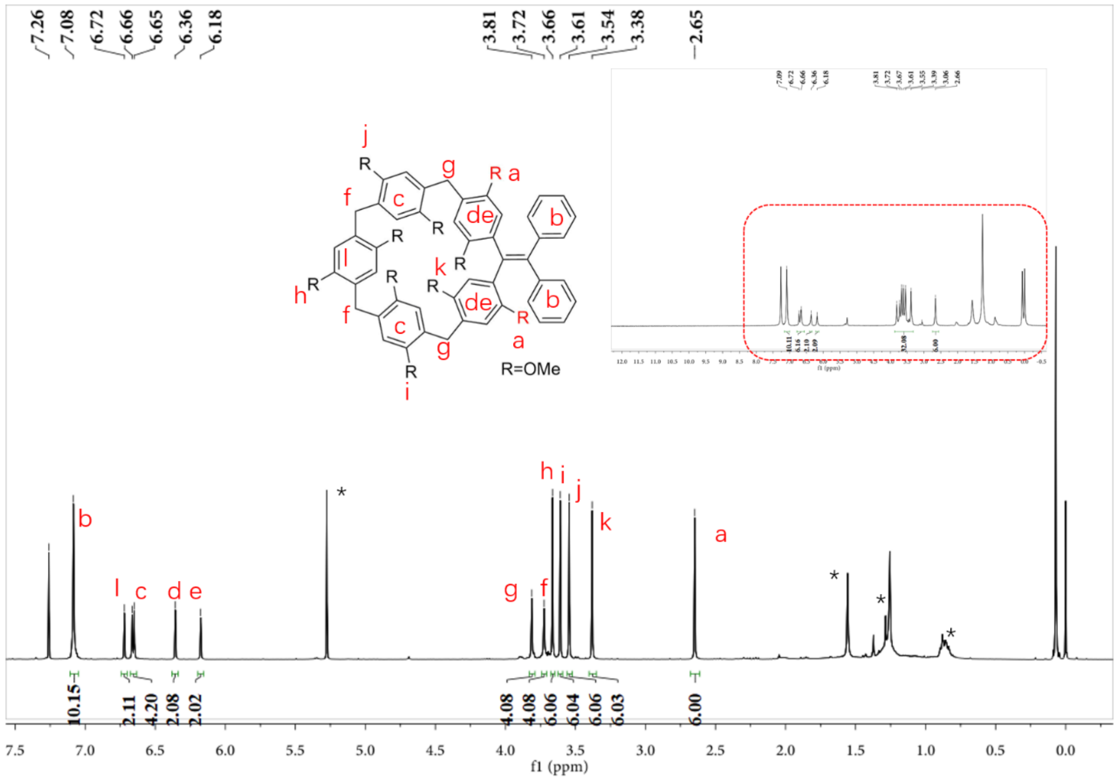

Figure S1. ^{1}H NMR spectrum of **DMP[5]-TPE** in $CDCl_3$.

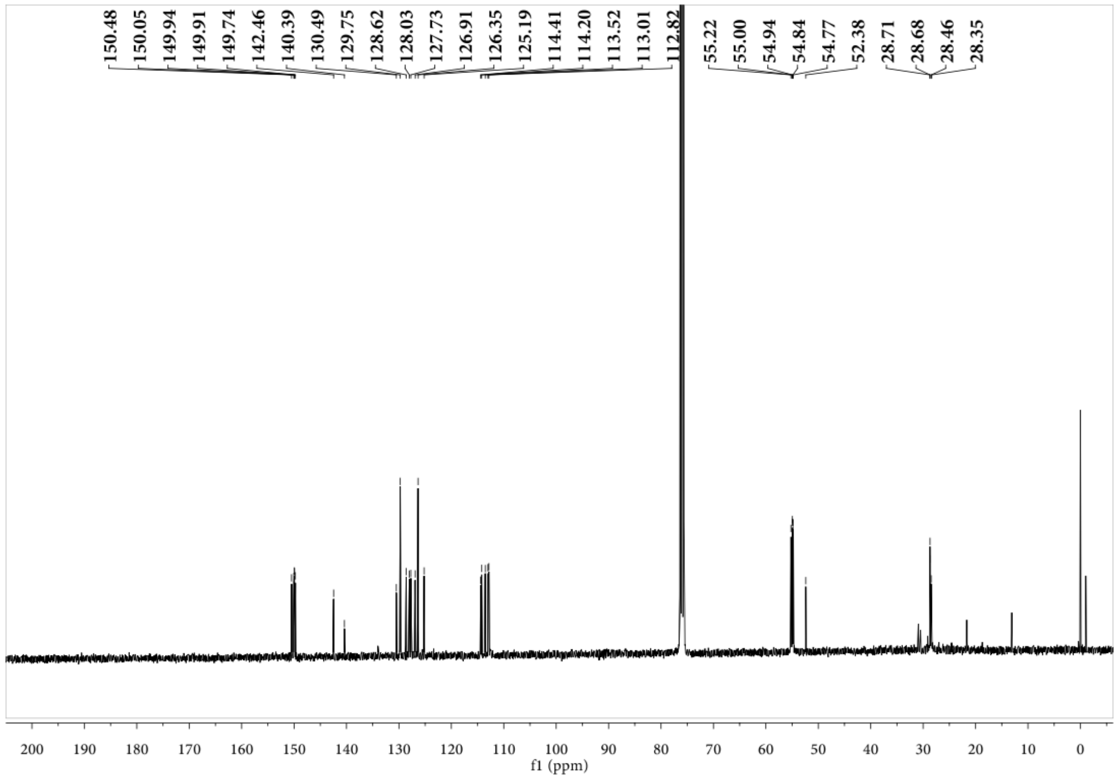

Figure S2. ^{13}C NMR spectrum of **DMP[5]-TPE** in $CDCl_3$.

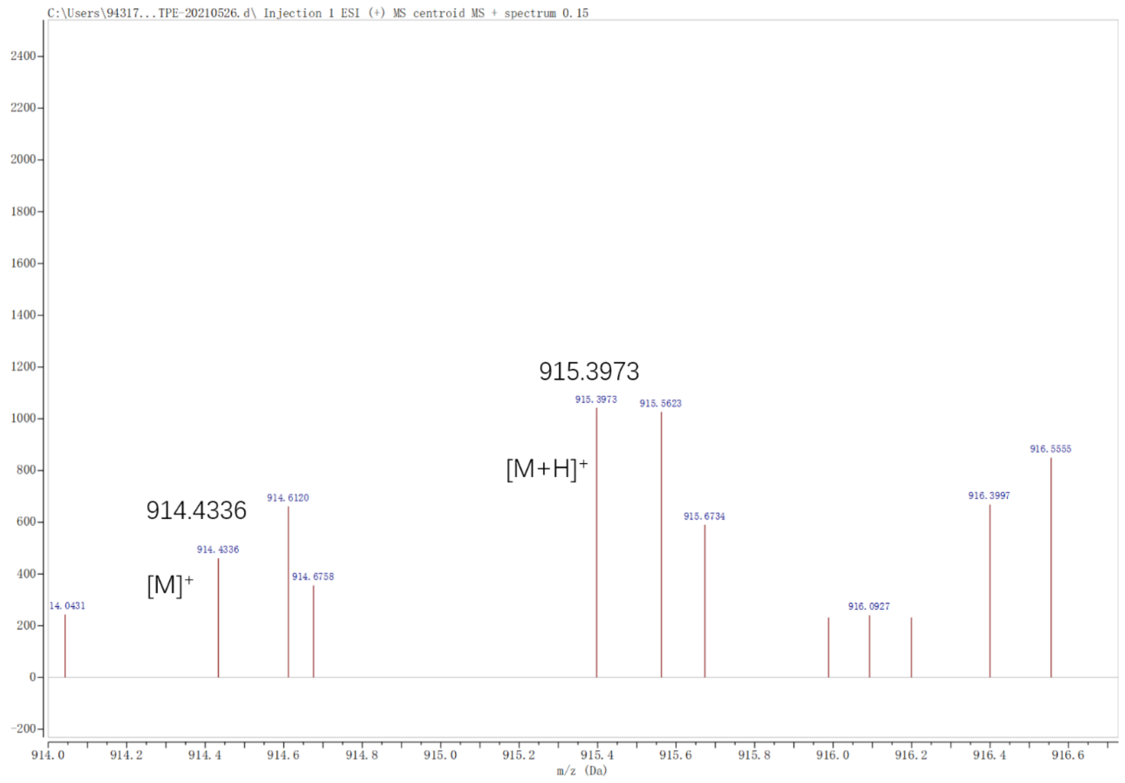

Figure S3. HRMS data for **DMP[5]-TPE** (Calculated $[M+H]^+$: $C_{58}H_{59}O_{10}^+$ = 915.4103, Found: 915.3973)

.

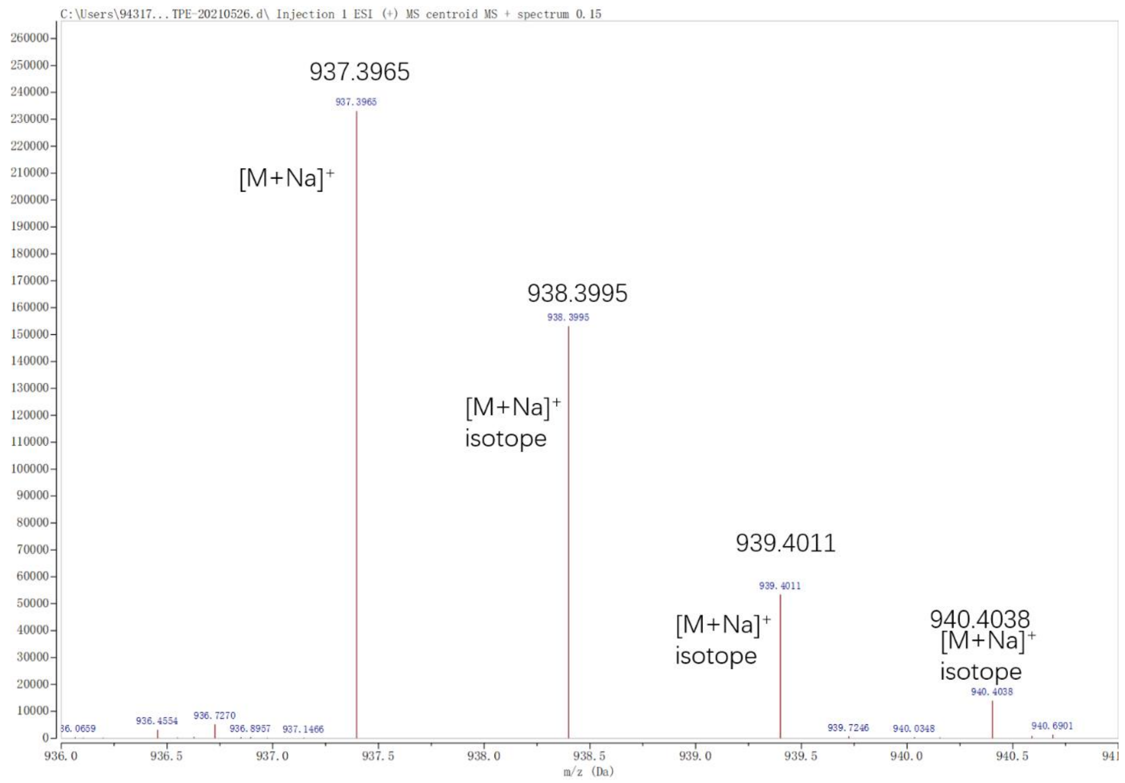

Figure S4. HRMS data for **DMP[5]-TPE** (Calculated $[M+Na]^+$: $C_{58}H_{58}NaO_{10}^+$ = 937.3922, Found: 937.3965)

3. Synthesis and characterization of **PCP[5]-TPE**

The synthesis of **PCP[5]-TPE** was similar to the **DMP[5]-TPE**. To a solution of $[1_5]$PCP (200 mg, 0.44 mmol) in anhydrous THF (1.5 mL), 2.4 M solution of *n*-butyllithium in hexane (0.92 mL, 2.22 mmol) was added at 0˚C under nitrogen. Afterwards, the reaction mixture was gradually brought back to room temperature (25˚C) and strirred 1 h. 4,4'-Dimethoxybenzophenone (646 mg, 2.67 mmol) was degassed and added to the reaction solution under nitrogen. The reaction temperature was warmed to 70˚C and stirred overnight. The recation quenched by adding H_2O (1 mL). Subsequently, the solvent was evaporated under vacuum, offering red-orange solid. The obtained solid was dissolved in CH_2Cl_2 (50 mL), and washed with 1N HCl (10 mL) and H_2O (2 ×30 mL)，dried over anhydrous Na_2SO_4 and concentrated.

The resulting solid was dissolved in toluene (60 mL), *p*-toluenesulfonic acid (190 mg) was added and the reaction mixture was refluxed for 6 h using a Dean-Stark apparatue. The toluene was evaporated under vacuum, offering oily liquid. The obtained oily liquid was dissolved in CH_2Cl_2 (50 mL), and washed with saturated Na_2CO_3 solution (20 mL) and H_2O (30 mL), dried over anhydrous Na_2SO_4 and concentrated. The concentrate was diluted with CH_2Cl_2 (1 mL) and slowly added dropwise to the vigorously stirred CH_3OH (50 mL). The solid immediately precipitated, suction filtrated, and dried to afford pale-yellow solid. Pure **PCP[5]-TPE** (284 mg, 95%) was obtained by column chromatography (PE/dichloromethane = 4/1, *v* / *v*). ^{1}H NMR (400 MHz, $CDCl_3$, 298 K) δ 7.03 (s, 4H), 6.91 (d, J = 54.7 Hz, 12H), 6.75 (s, 1H), 6.71 (s, 8H), 6.64 (d, J = 6.7 Hz, 4H), 3.81 (s, 4H), 3.75 (s, 4H), 3.73 (s, 6H).. ^{13}C NMR (101 MHz, $CDCl_3$, 298 K) δ 157.96, 142.11, 139.66, 139.39, 139.22, 136.35, 132.48, 130.99, 129.18, 128.65, 128.43, 127.79, 113.00, 55.09, 41.47, 41.18. Anal. Calcd. for $C_{50}H_{42}O_2$: H, 6.27; C, 88.99, Found: H, 6.28; C, 89.13.

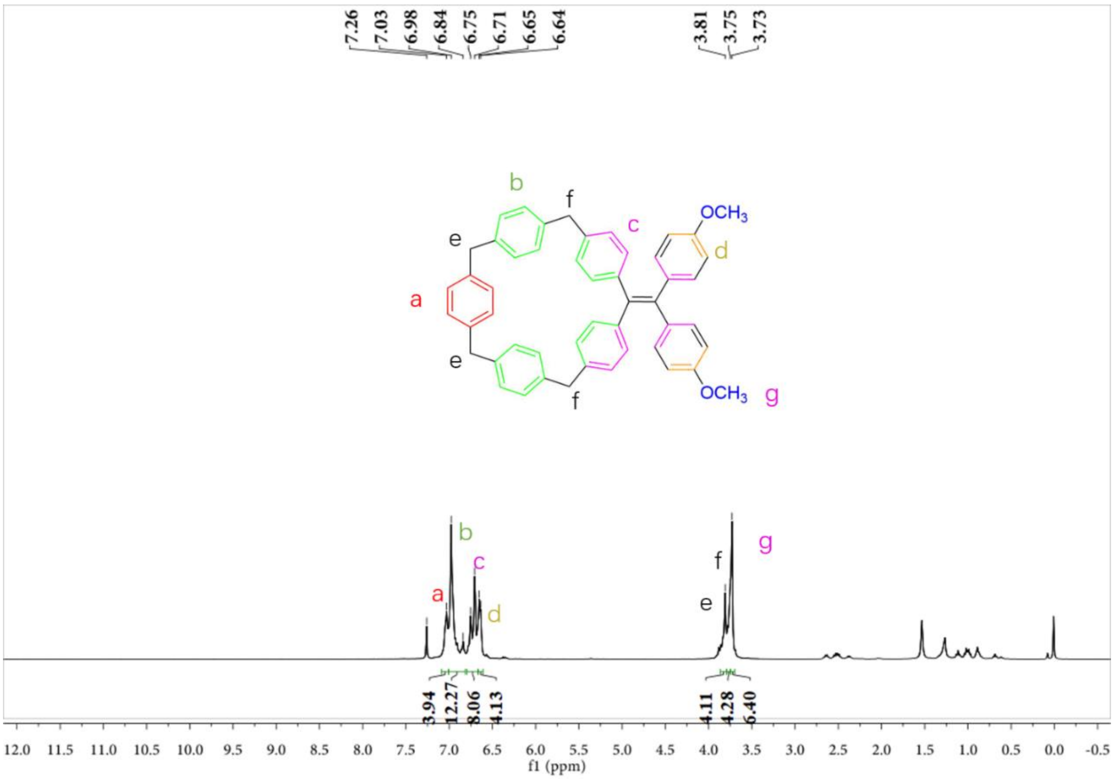

Figure S5. ^{1}H NMR spectrum of **PCP[5]-TPE** in $CDCl_3$.

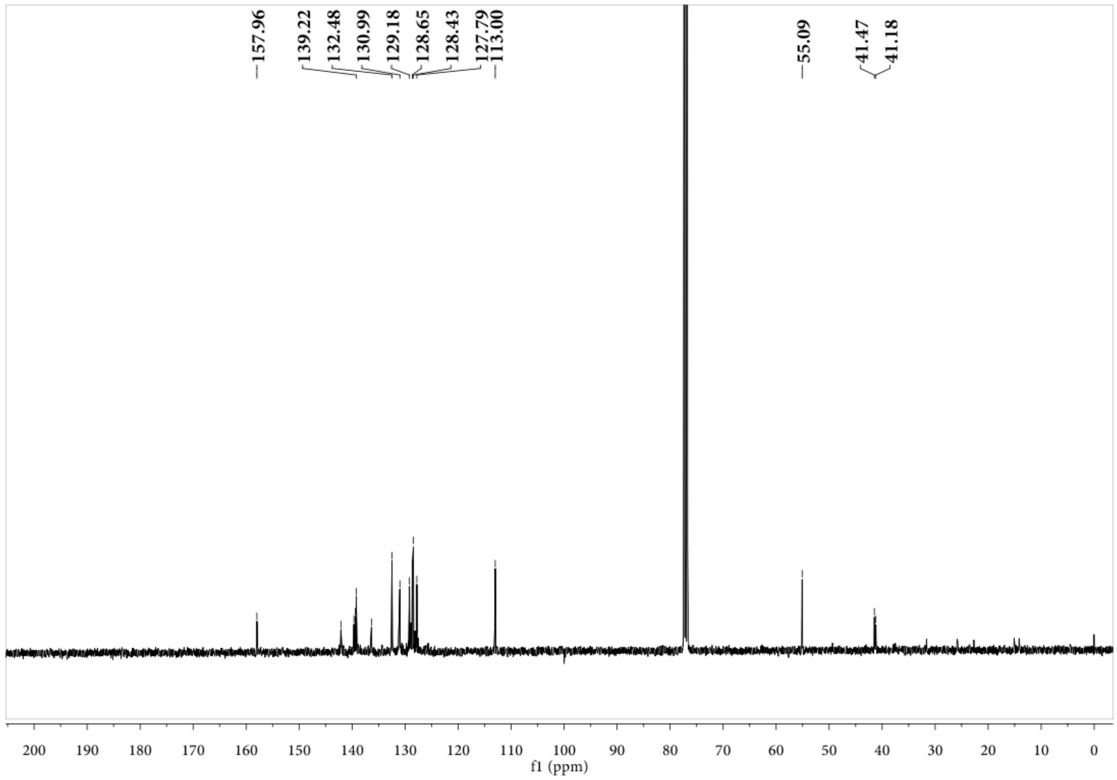

Figure S6. ^{13}C NMR spectrum of **PCP[5]-TPE** in $CDCl_3$.

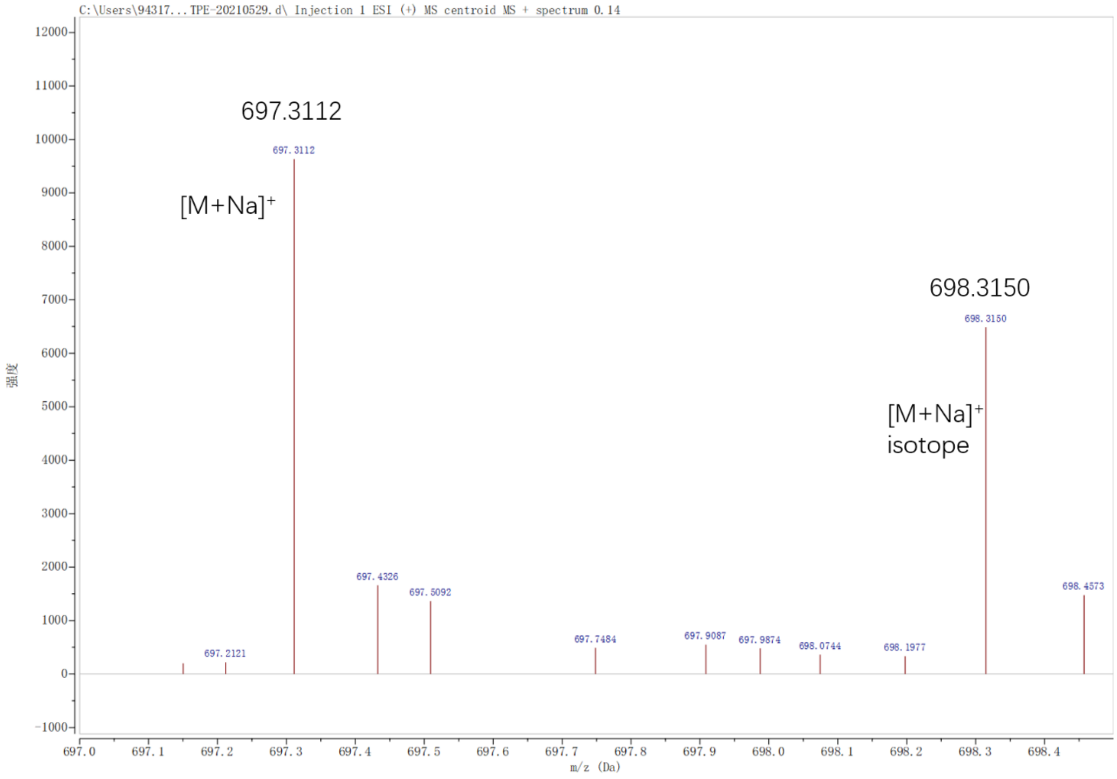

Figure S7. HRMS data for **PCP[5]-TPE** (Calculated $[M+Na]^+$: $C_{50}H_{42}NaO_2^+$ = 697.3077, Found: 697.3122)

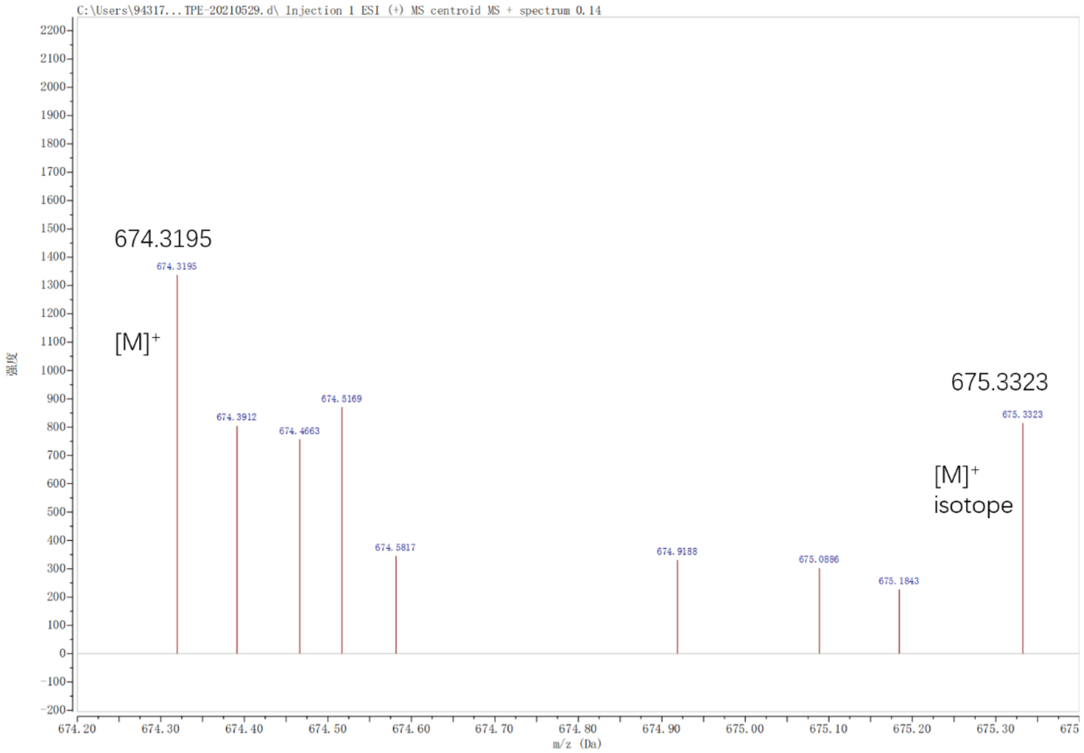

Figure S8. HRMS data for **PCP[5]-TPE** (Calculated $[M]^+$: $C_{50}H_{42}O_2^+$ = 674.3179, Found: 674.3195)

4. X-ray crystallography data of PCP[5]-TPE

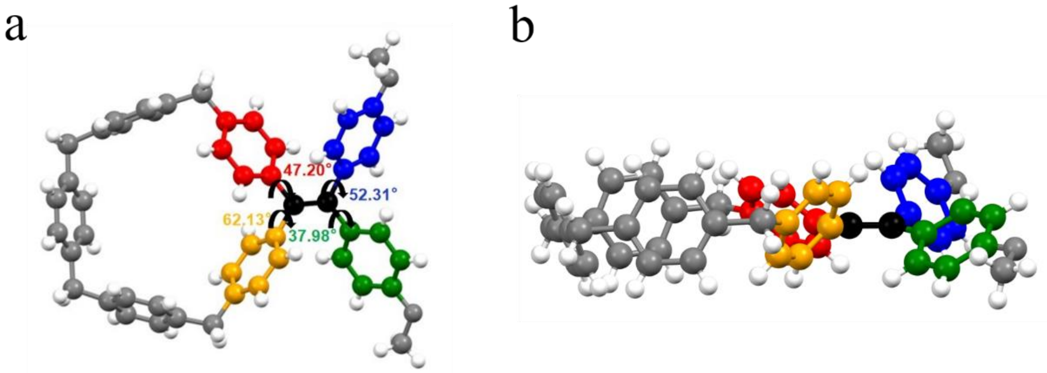

Figure. S9. Top (a) and side view (b) of crystal structure **PCP[5]-TPE**

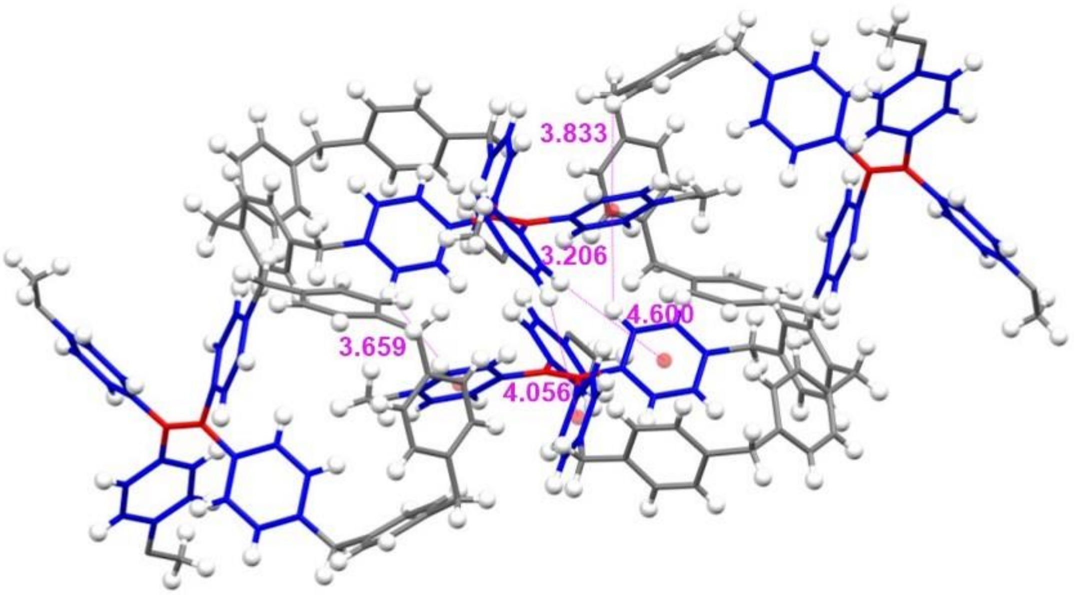

Figure. S10. Molecular packing of **PCP[5]-TPE**

5. X-ray crystallography data of DMP[5]-TPE

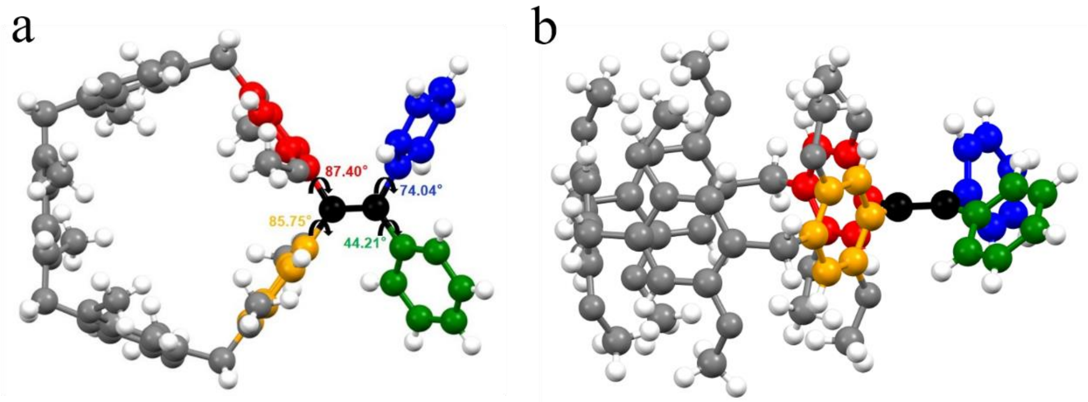

Figure. S11. Top (a) and side view (b) of crystal structure DMP[5]-TPE. Hexane solvent molecules are omitted for clarity.

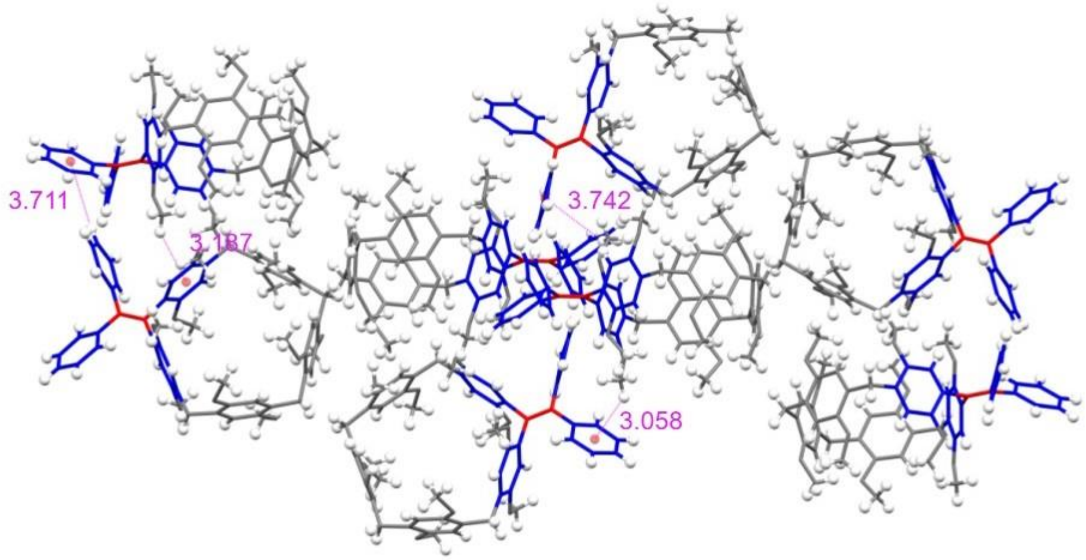

Figure. S12. Molecular packing of DMP[5]-TPE. Hexane solvent molecules are omitted for clarity.

6. Fluorescence spectra of DMP[5]-TPE and PCP[5]-TPE

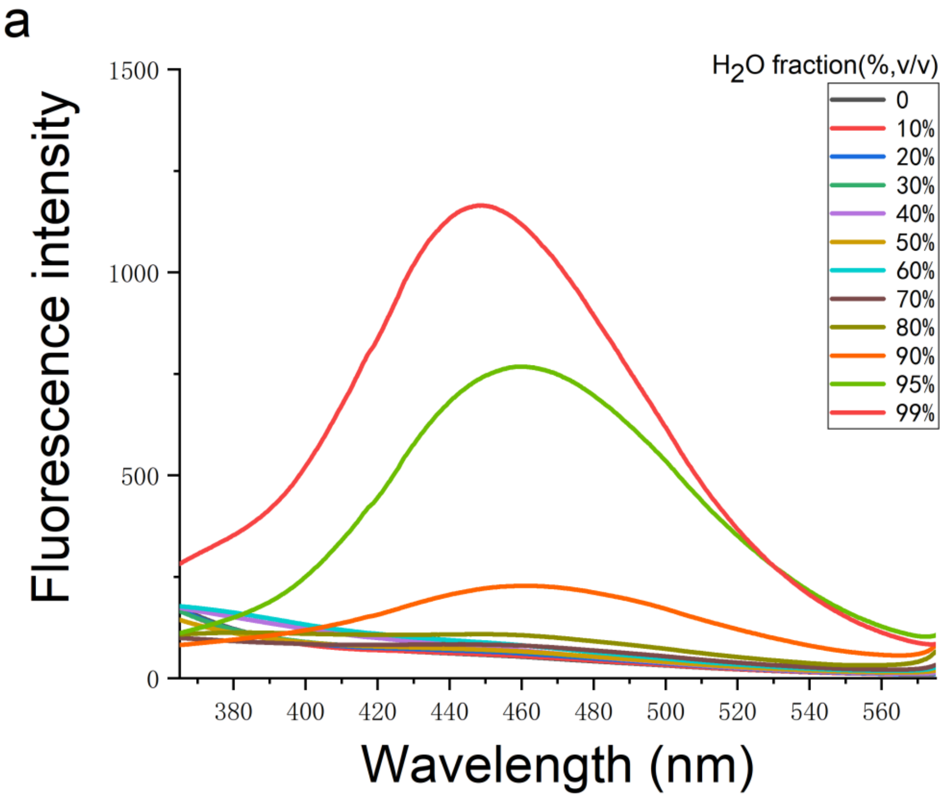

Figure S13. Fluorescence spectra of **DMP[5]-TPE** (1 x 10^{-5} M)

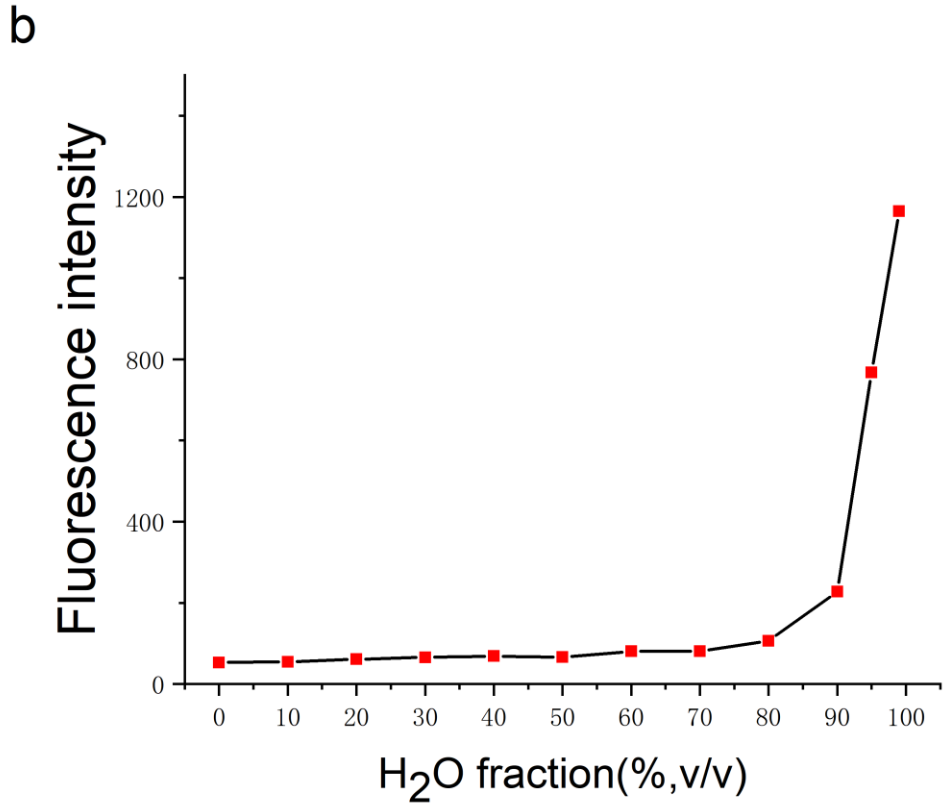

Figure S14. Plot of the highest fluorescence intensity in different THF/H_2O mixed solution of **DMP[5]-TPE**.

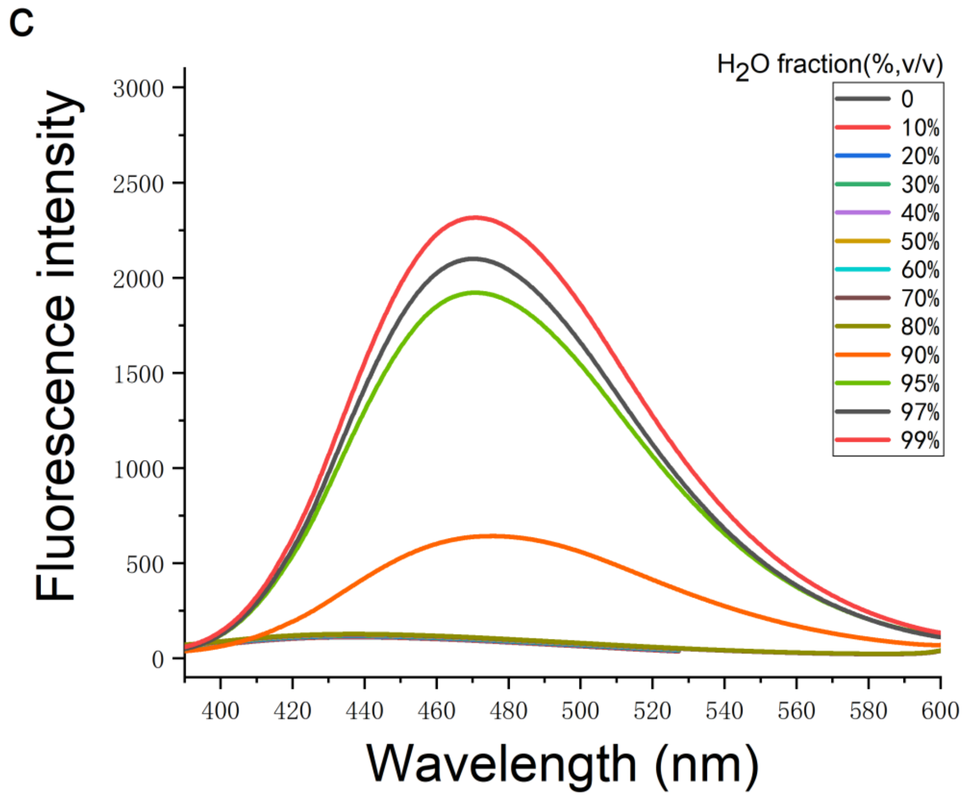

Figure S15. Fluorescence spectra of **PCP[5]-TPE** (1 x 10^{-5} M)

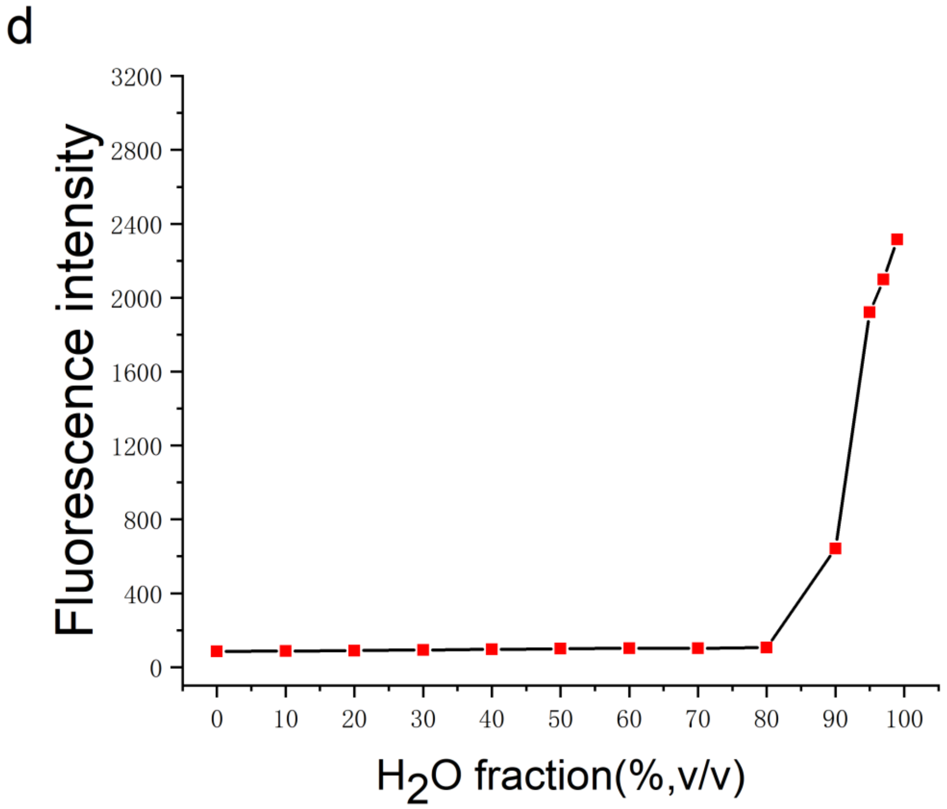

Figure S16. Plot of the highest fluorescence intensity in different THF/H_2O mixed solution of **PCP[5]-TPE**.

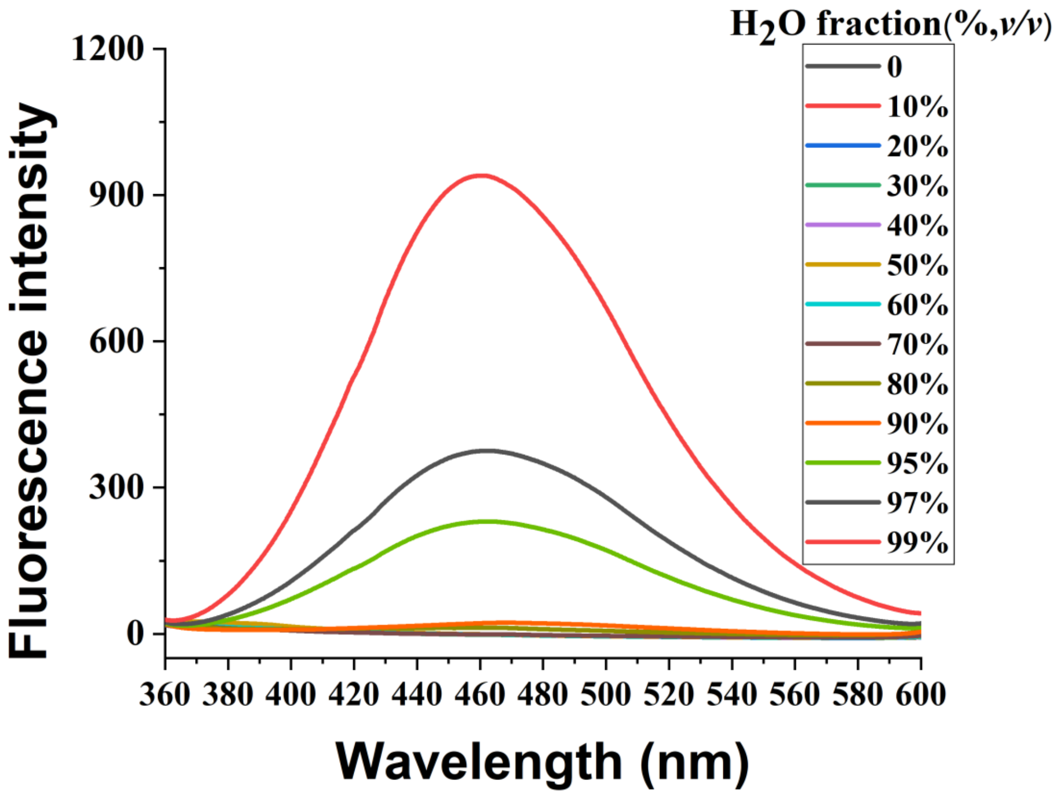

Figure S17. Fluorescence spectra of **PC[5]-TPE** (1 x 10^{-5} M)

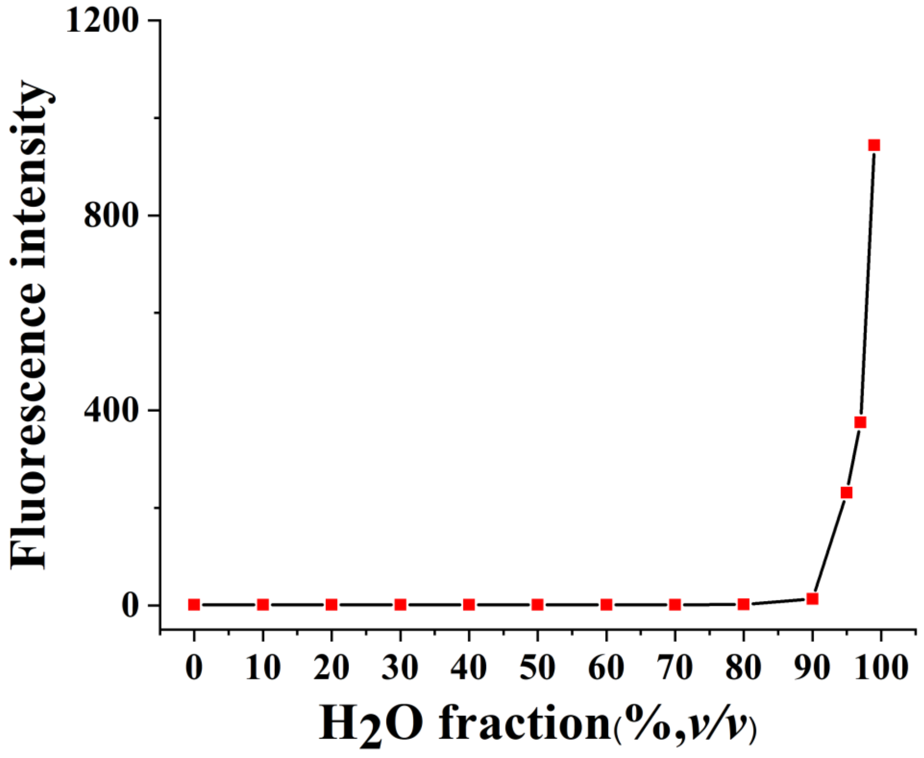

Figure S18. Plot of the highest fluorescence intensity in different THF/H_2O mixed solution of **PCP[5]-TPE**.

7. Binding experiment with 1,4-dicyanobutane of **DMP[5]-TPE** and **DMP[5]**

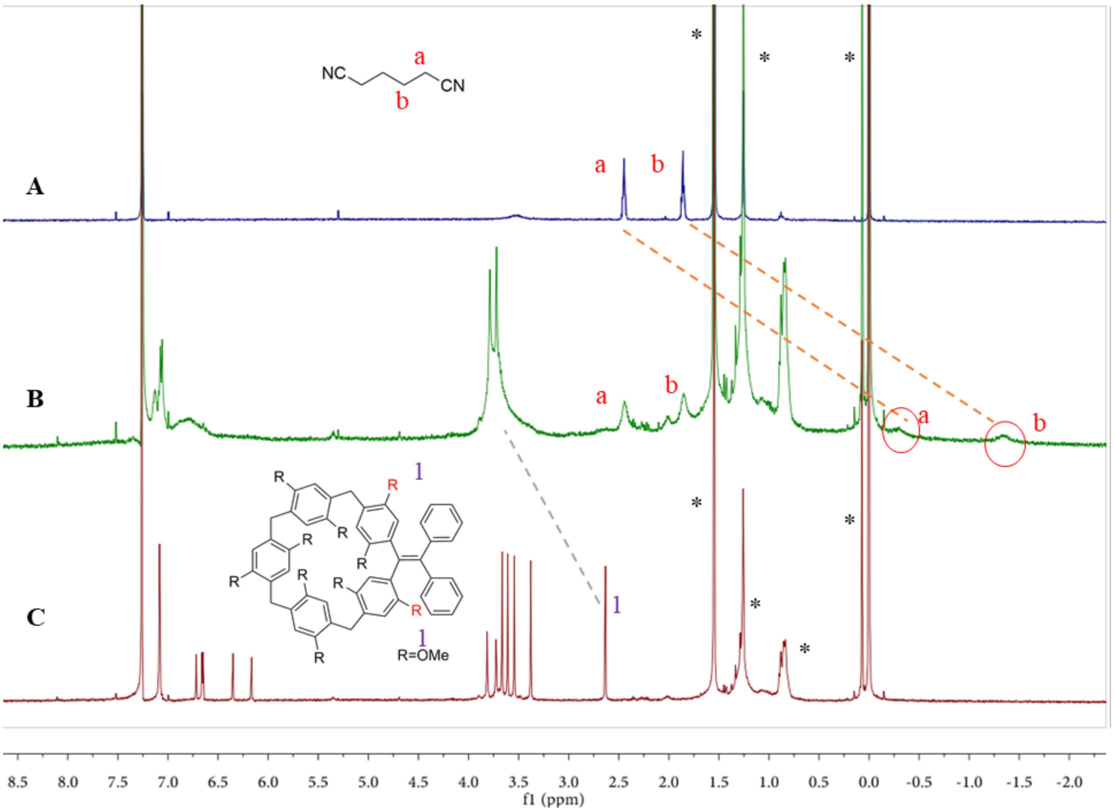

Figure S19. (A) ^{1}H NMR spectra of DCB (1 mM), (B) Partial ^{1}H NMR spectra of mixed solution of **DMP[5]-TPE** (1 mM) and DCB (1 mM), (C) ^{1}H NMR spectra of **DMP[5]-TPE** (1 mM). * = solvent.

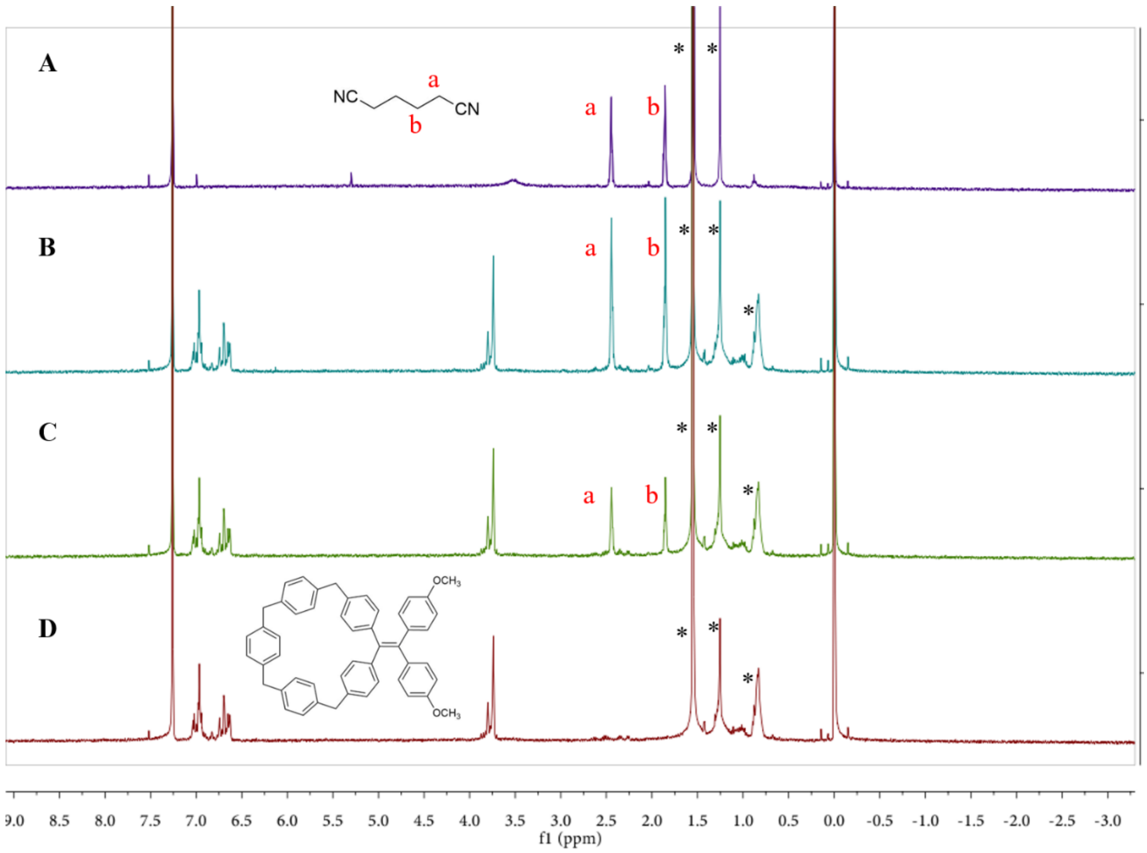

Figure S20. (A) ^{1}H NMR spectra of DCB (1 mM), (B) Partial ^{1}H NMR spectra of mixed solution of **PCP[5]-TPE** (1 mM) and DCB (1 mM), (C) Partial ^{1}H NMR spectra of mixed solution of **PCP[5]-TPE** (1 mM) and DCB (2 mM), (D) ^{1}H NMR spectra of **PCP[5]-TPE** (1 mM). * = solvent.

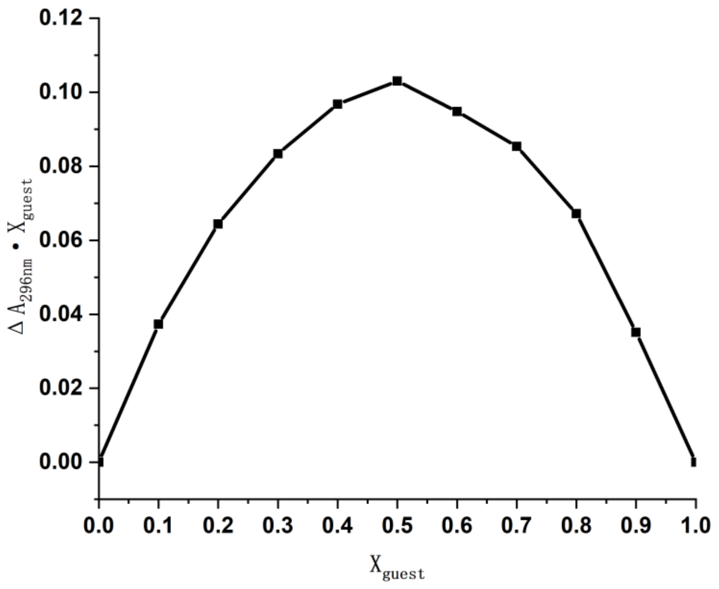

Figure S21. Job plot showing the 1: 1 stoichiometry of the complex between **DMP[5]-TPE** and DCB in $CHCl_3$ by plotting the Δδ in absorbance of the 280nm observed by uv-vis against

the mole fraction of guest (X_{guest}). ([host] + [guest] = 2×10^{-5} M.

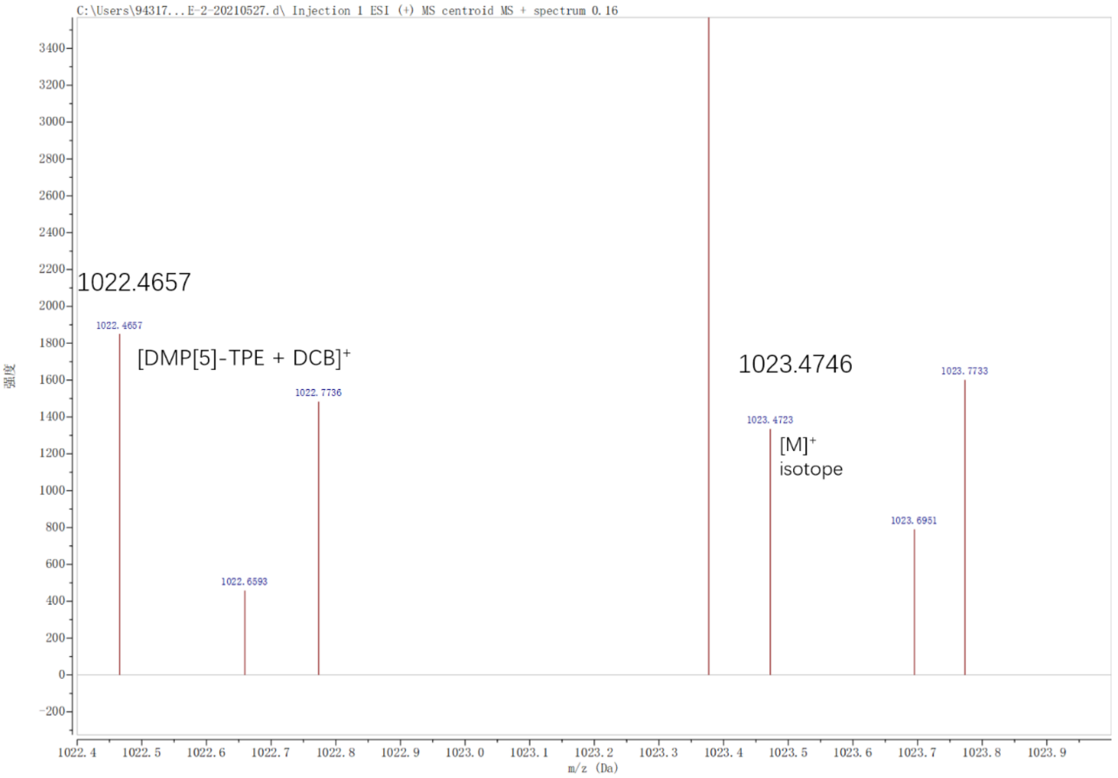

Figure S22. ESI-MS spectrum of the host−guest complex formed between **DMP[5]-TPE** and DCB. (Calculated [**DMP[5]-TPE** + DCB]$^+$: $C_{64}H_{66}N_2O_{10}^+$ = 1022.4712, Found: 1022.4657)

To determine the binding constant, uv-vis titration experiments were carried out in $CHCl_3$ which had a constant concentration of host (1×10^{-5} M) and varying concentration of guest. A nonlinear curve-fitting method was applied to calculate the association constant for the complexation between host and guest, which was based on the following equation:

Δδ=(P1/0.00001)*(0.5*x+0.5*(0.00001+1/P2)-(0.5*(x^2+(2*x*(1/P2-0.00001))+(1/P2+0.00001)^2)^0.5))

Where Δδ is the absorbance of host and guest mixture at 281 nm, P1 is the fixed initial concentration of the host, and P2 is the varying concentrations of the guest.

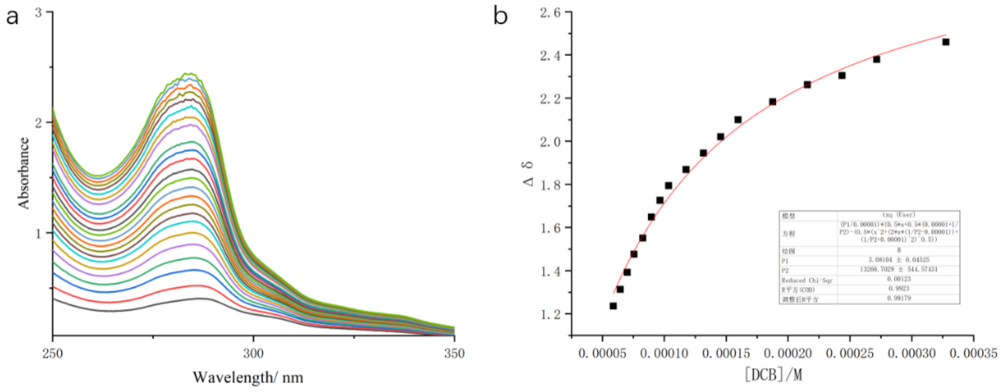

Figure S23. (a) The absorbance of **DMP[5]-TPE** and DCB mixture. (b)The Red line was obtained from the nonlinear curve-fitting (K_a = ((1.33± 0.05) × 10^4 M^{-1}, R^2 =0.9923)

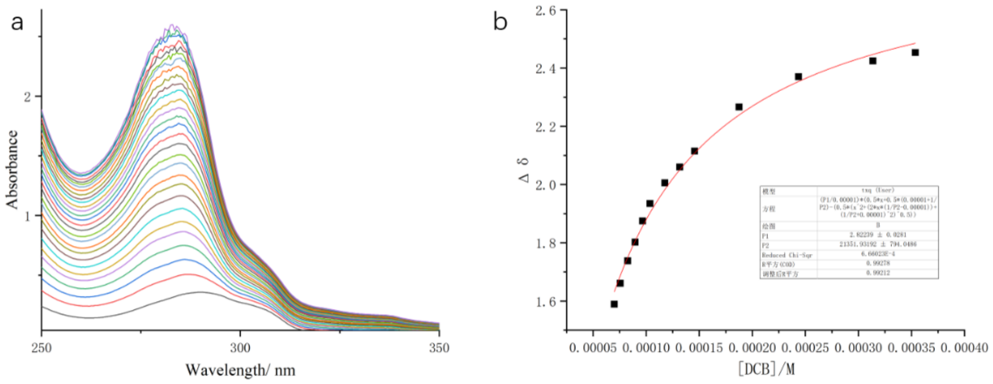

Figure S24. (a) The absorbance of DMP[5] and DCB mixture. (b)The Red line was obtained from the nonlinear curve-fitting (K_a = ((2.14± 0.08) × 10^4 M^{-1}, R^2 =0.9928)

Host	Association constant with DCB
DMP[5]	2.14± 0.08) × 10^4 M^{-1}
DMP[5]-TPE	1.33± 0.05) × 10^4 M^{-1}
PCP[5]-TPE	No complexation

Table S1. Association constant for DMP[5], DMP[5]-TPE and PCP[5]-TPE.

1. Liu, P.; Li, Q.; Zeng, H.; Shi, B.; Liu, J.; Huang, F., $[1_5]$Paracyclophane and $[1_6]$paracyclophane: facile syntheses, crystal structures and selective complexation with cesium cations in the gas phase. *Organic Chemistry Frontiers* **2019,** 6, (3), 309-312.
